# Enhanced Mesophilic Degradation of Rice Straw by Microbial Consortium SXJG15 Through Coordinated Enzymatic Activity and Community Reshaping

**DOI:** 10.3390/microorganisms13122707

**Published:** 2025-11-27

**Authors:** Zhen Zhang, Mohammad Shafiqul Islam, Muhammad Noman, Zhongna Hao, Rongyao Chai, Haiping Qiu, Jing Wang, Yingying Cai, Yanli Wang, Jiaoyu Wang

**Affiliations:** 1 State Key Laboratory of Agricultural Products Safety, Key Laboratory of Agricultural Microbiome of Zhejiang Province, Institute of Plant Protection and Microbiology, Zhejiang Academy of Agricultural Sciences, Hangzhou 310021, China; zhangz@zaas.ac.cn (Z.Z.); shafiqmohammadst@gmail.com (M.S.I.); m.noman@zju.edu.cn (M.N.); haozhongna1999@sina.com (Z.H.); rychai@sina.com (R.C.); qiuhping@163.com (H.Q.); wj9311@163.com (J.W.); caiyy@zaas.ac.cn (Y.C.); 2 College of Biotechnology and Bioengineering, Zhejiang University of Technology, Hangzhou 310014, China

**Keywords:** CAZyme profiling, metagenomics, microbial consortium, mesophilic fermentation, rice straw biodegradation

## Abstract

The sustainable utilization of rice straw is challenged by its recalcitrant lignocellulosic structure, especially under low-to-moderate field temperatures. In this study, a novel microbial consortium (SXJG15) mainly containing *Sphingobacterium*, *Azospirillum*, and *Pseudomonas* was enriched from overwintering rice stubble in Zhejiang, China, and evaluated for its rice straw degradation efficiency at 25 °C. Over an 18-day cultivation period, SXJG15 achieved a 52.5% degradation of total rice straw, including 60.2% cellulose, 76.3% hemicellulose, and 40.7% lignin. High extracellular enzymatic activities, including cellulases (up to 80.3 U/mL) and xylanases (up to 324.8 U/mL), were observed during the biodegradation process. *16S rRNA* gene sequencing and metagenomics analyses revealed a succession of dominant taxa, including *Sphingobacterium*, *Azospirillum*, and *Cellulomonas*. Further, CAZy annotation indicated that the SXJG15 enzyme system was rich in glycoside hydrolases (42.7%) and glycosyltransferases (34.2%), demonstrating its high potential for lignocellulose degradation. This study uniquely demonstrates the mesophilic (moderate temperature 25 °C) efficiency of SXJG15 in lignocellulose breakdown, provides new insights into the microbial mechanisms of straw decomposition, and lays a foundation for bioenergy and soil fertility applications for developing a sustainable agriculture system.

## 1. Introduction

Rice straw, as the main agricultural residue after rice harvest, is one of the largest biomass resources in global agricultural production. The global production of rice straw reaches 800–1000 million tons annually, contributing significantly to agricultural waste accumulation [1]. With the increasing intensiveness of rice cultivation and the shortening of the growth cycle, the production of rice straw continues to rise, and the problem of regional rice straw surplus has become increasingly prominent. This surplus has directly led to the widespread existence of improper treatment methods such as field burning, which not only causes serious air pollution, but also affects the ecological balance of the soil and wastes precious biomass resources [2].

From a resource-recycling perspective, returning straw to the field is widely practiced in China, where it enhances soil organic matter and fertility [3]. However, it should be pointed out that the main structural components of rice straw (cellulose, hemicellulose, and lignin) possess highly stable chemical properties and decompose slowly in the natural environment [4]. This slow decomposition process makes it difficult to quickly improve soil fertility after large-scale return to the field. Instead, it may cause problems such as imbalance of soil microbial communities and the breeding of pests and diseases [2,5]. This limited degradability hinders timely nutrient cycling and may even contribute to microbial imbalance and crop diseases [2,5]. Therefore, developing efficient straw degradation technology and improving its resource conversion efficiency has become an important issue in promoting sustainable agricultural development.

Efficient degradation of rice straw can be achieved through physical, chemical, and biological methods [6,7]. Among these, biological degradation at low to moderate temperatures (15–25 °C) is generally slower due to reduced microbial activity, necessitating the use of specialized consortia. Nevertheless, augmented biological treatment technologies, such as microbial fermentation and enzymatic hydrolysis, remain the most promising due to their environmental compatibility, low energy demand, and efficient lignin degradation capability [8]. Hydrolysis under mesophilic conditions (~25 °C) further offers a sustainable advantage by minimizing external energy consumption while maintaining effective enzymatic activity, providing an energy-efficient alternative to conventional high-temperature processes [7]. Studies have shown that the use of cellulolytic microorganisms to degrade rice straw has a significant effect.

However, in practical applications, microbial degradation efficiency is highly dependent on environmental parameters such as pH, temperature, humidity, and straw structural characteristics, which may even cause differences in ability of strains under different conditions. Therefore, the selection and breeding of efficient and adaptable degradation strains or bacterial communities remain the focus and difficulty of research in this field.

Therefore, it is urgent to find effective rice-straw decomposing microbial communities. While recent studies have shown that psychrophilic consortia (e.g., LTF-27 at 15 °C) [8] and thermophilic systems (e.g., StrBsyn at 37 °C) [9] efficiently decompose lignocellulose-rich biomass, robust rice straw biodegradation systems at moderate-to-low temperatures (20–25 °C) remain underdeveloped. SXJG15 addresses this gap by combining *Sphingobacterium* (cellulase/pH regulation) and *Cellulomonas* (hemicellulase) for rapid 25 °C decomposition. Rapid decomposition is essential to mitigate root rot risks from slow-decaying straw [5].

In this study, a microbial consortium with stable rice straw degradation performance was screened from overwintering rice straw in Zhejiang, China, through continuous enrichment culture. This study investigates the fermentation performance, structure, and kinetics of microbial communities, as well as the characterization of their multi-species enzyme systems. The microbial communities established in this study provide a valuable platform for further exploration of downstream applications in agriculture waste biodegradation and bioenergy, while addressing the urgent need for robust straw-degrading consortia adaptable to mesophilic conditions.

## 2. Materials and Methods

### 2.1. Materials

The rice straw used in this study was collected from the farm of Zhejiang Academy of Agricultural Sciences, Hangzhou, China, air-dried, cut into small segments (3–5 cm), and pretreated with 1% NaOH (*w*/*v*) solution at room temperature (23 ± 1 °C) for 24 h according to the method of Chen et al. [10]. All chemical reagents used were of analytical grade and purchased from Shanghai Sangon Biotechnology Co., Ltd. (Shanghai, China).

### 2.2. Enrichment of Straw-Degrading Microorganisms

The overwintering rice stubble was collected from the winter idle farmland in Shaoxing City, Zhejiang Province, China, and then air-dried and ground using a household stainless steel grinder. Three grams of crushed winter rice stubble sample was spread evenly on sterile, starch-free filter paper, placed over a moist culture substrate, and incubated at 25 °C until transparency indicated cellulose degradation [11,12]. The bacterial moss was scraped on the above culture medium and resuspended in 20 mL of sterile water. 5 mL was taken and put into a conical flask containing 100 mL PCS culture medium [8] containing pretreated rice straw (10 g L^−1^), tryptone (5 g L^−1^), yeast extract (1 g L^−1^), NaCl (5 g L^−1^), and CaCO_3_ (2 g L^−1^). The addition of starch-free filter paper strips indicated the degradation effect of cellulose. The SXJG15 microbial consortium was obtained through repeated sub-culturing under static, facultative anaerobic conditions at 25 °C. It was cryopreserved in 50% glycerol at −80 °C.

### 2.3. Determination of the Degradation Rate of Rice-Straw-Degrading Bacteria

SXJG15 was inoculated into PCS culture medium [8] at 5% (*v*/*v*) and incubated at 25 °C under aerobic conditions, with the cultures maintained on a shaker at 150 rpm to ensure consistent mixing and aeration [8]. Samples were taken at 0, 1, 3, 6, 9, 12, 15, and 18 days after inoculation to determine the pH value, straw degradation rate, and straw-degradation-related enzyme activities at different culture stages. An uninoculated culture medium was used as a blank control (*n* = 3). Supernatants were collected via centrifugation at 12,000 rpm for enzymatic assays. Cellulose activity was determined using the anthrone colorimetric method [13], where 1 U of cellulase activity = 1 μmol glucose released/min/mL; xylanase activity was determined using the DNS method [14], where 1 U of xylanase activity = 1 μmol xylose released/min/mL. Lignin peroxidase activity was determined using the Azure B method [15], and manganese peroxidase activity was measured using the guaiacol method [16]. A control was used, which eliminates the influence of the reducing sugars in the samples. The substrate, xylans, has substrate specificity for xylanase, and other enzymes such as cellulase cannot catalyze the decomposition of this substrate to produce reducing sugars.

The residual rice straw in the triangular bottle was collected with a 100-mesh filter, and the rice straw was thoroughly washed with clean water, dried at 105 °C for 8 h, and weighed to determine the weight loss. The dried sample was crushed and sieved with a 40-mesh sieve. The Van Soest method [17] was used to accurately determine the lignin, cellulose, and hemicellulose contents in rice straw, and the corresponding degradation rates were calculated. The degradation rate (%) was calculated as follows:

Degradation rate (%) = ((W_0_ − W_t_) /W_0_) × 100

where W_0_ is the initial dry weight (g) of the substrate and W_t_ is the dry weight (g) after incubation at time t.

Cellulose, hemicellulose, and lignin contents were quantified by the Van Soest [17] detergent fiber method (NDF/ADF/ADL). Component masses were calculated from the detergent fractions using the following relationships:

[Hemicellulose (g) = NDF − ADF] [Cellulose (g) = ADF − ADL] [Lignin (g) = ADL]

Component-specific degradation rates were determined by applying the degradation rate equation above to the initial and residual masses of each component.

### 2.4. DNA Extraction, PCR Amplification, and Microbial Community Analysis

The culture fluid of the SXJG15 consortium was taken after 1, 3, 6, 9, 12, 15, and 18 days of culture to extract genomic DNA for *16S rRNA* gene sequencing [18]. The samples cultured for 18 days were collected for metagenomic sequencing. Total Genomic DNA was extracted using the chlorobenzene method and amplified using universal primers 338F (5′-ACTCCTACGGGAGGCAGCA-3′) and 806R (5′-GGACTACHVGGGTWTCTAAT-3′) targeting the bacterial V3-V4 region of the *16S rRNA* gene [8]. All procedures were carried out by Gene Denovo Biotechnology Co. Ltd. (Guangzhou, China). The raw reads were submitted to the NCBI with the accession No. PRJNA1298514 for the microbial community and No. PRJNA1298566 for the metagenomics data.

### 2.5. Statistical Analysis

The data on pH value, lignin degradation enzyme activity, and lignocellulose degradation rate were analyzed by Graphpad 9.0 software. The principal component analysis (PCA) method was used to evaluate the microbial community structure based on the Bray–Curtis distance [19]. Metastats statistical algorithm in Mothur software (Version 1.48.1) was used to perform pairwise tests on the differences in the number of sample sequences at the genus level (i.e., relative abundance) [20,21]. Microbial species composition and functional genes of bacteria were annotated according to the KEGG (http://www.genome.jp/kegg, accessed on 26 August 2024) database [22] and the NCBI-NT database (http://www.ncbi.nlm.nih.gov/, accessed on 26 August 2024), where all annotation levels were considered for a comprehensive evaluation. The Unigene sequence set was uploaded to the dbCAN database (a database for automatic annotation of carbohydrate-active enzymes) (https://bcb.unl.edu/dbCAN2, accessed on 30 August 2024) for functional annotation, and the returned annotation results were summarized [23] and counted to obtain the annotation results at each level and the corresponding abundance information.

## 3. Results and Discussion

### 3.1. Changes in pH

**Figure 1:**
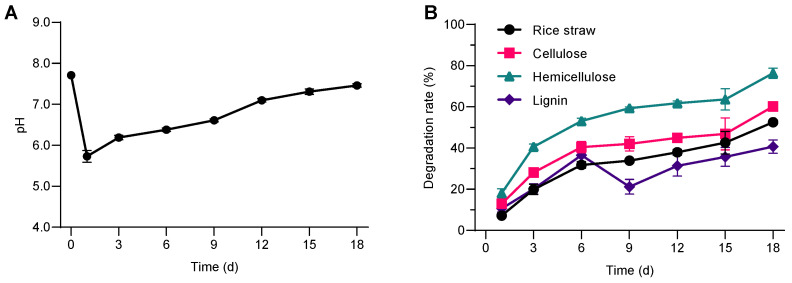
Degradation characteristics of rice straw by bacterial colony SXJG15. (**A**). pH; (**B**) biodegradation of rice straw and its components. All values represent mean ± standard deviation (*n* = 3).

As shown in (Figure 1A), as the culture time increased, the pH value rapidly decreased from the initial 7.71 to 5.73 on the first day. The pH value then slowly increased, returning to 7.10 on the 12th day, and then remained stable until the end of the experiment. The stabilization of pH after this point is consistent with findings by Zhao et al. (2014) [24], who observed that after initial acidification, microbial communities involved in lignocellulose degradation tend to metabolize these acids, leading to a neutralization of the medium. Similar observations were made by Liu et al. (2021) [25], who reported a similar pH pattern during the microbial degradation of rice straw, which was linked to both the production and subsequent utilization of organic acids during the degradation process. This pattern is consistent with the conversion of lignocellulosic biomass into organic acids during early microbial activity, followed by consumption of those acids, resulting in a decrease in the pH value in the early stages of rice straw degradation [26,27,28]. A gradual increase in pH was observed during the incubation period, which may reflect shifts in microbial metabolism and substrate utilization. However, further quantification of soluble metabolites such as volatile fatty acids (VFAs) and soluble chemical oxygen demand (sCOD) would be required to clarify the underlying mechanisms.

### 3.2. Changes in the Degradation Rate of Rice Straw

Rice straw contains 32–47% cellulose, 19–27% hemicellulose, and 5–24% lignin [29]. As the consortium was cultivated, the degradation of rice straw, cellulose, hemicellulose, and lignin gradually increased over time (Figure 1B). After 1 day of cultivation, the straw degradation rate was 7.2%, and then gradually increased to 52.5% after 18 days of cultivation. The degradation trends of cellulose, hemicellulose, and lignin were basically consistent with the degradation of straw. After one day of cultivation, the degradation rates of cellulose, hemicellulose, and lignin were 13.0%, 18.2%, and 10.6%, respectively.

On the 18th day, the degradation rates were 60.2% for cellulose, 76.3% for hemicellulose and 40.7% for lignin, respectively. The above results show that at 25 °C, the bacterial consortium SXJG15 can effectively degrade hemicellulose, cellulose, and lignin in rice straw. Hemicellulose degraded more rapidly than cellulose or lignin, likely due to its lower structural complexity and accessibility (Figure 1B). SXJG15’s 52.5% degradation at 25 °C/18 days outperforms anaerobic consortium StrBsyn (48.9% at 37 °C/14 days) [9] and approaches psychrophilic LTF-27 (55.8% at 15 °C/30 days) [8], demonstrating unmatched speed in mesophilic (moderate temperature 25 °C) conditions. This pattern of hemicellulose being more labile than cellulose is consistent with its lower degree of polymerization, amorphous structure, and greater enzymatic accessibility reported in the literature.

### 3.3. Changes in the Activities of Lignocellulose-Degrading Enzymes

**Figure 2:**
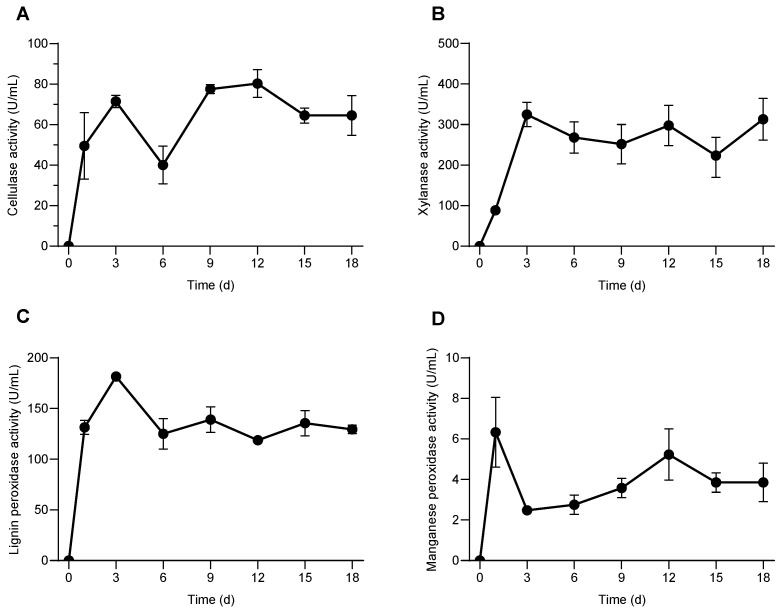
Effect of the consortium SXJG15 on the activities of enzymes related to rice straw degradation. (**A**) Cellulase; (**B**) xylanase; (**C**) lignin peroxidase; (**D**) manganese peroxidase. All values represent mean ± standard deviation (*n* = 3).

Microorganisms mainly degrade lignocellulose by secreting extracellular enzymes. Cellulose, xyloses, lignin peroxidase, manganese peroxidase, and other enzymes are known to participate in the enzymatic hydrolysis of lignocellulose [30,31]. The above enzymes showed high activity in the process of rice straw degradation by the consortium SXJG15. The enzymatic activity of cellulose was 49.5 U/mL on the first day after inoculation, reached a peak of about 80.3 U/mL on the 12th day, and then began to decline, but the enzyme activity was still 64.5 U/mL on the 18th day (Figure 2A). This pattern aligns with previous findings where cellulase activity peaks after several days of incubation and then declines but maintains functional levels throughout degradation [30,32].

The enzymatic activity of xylanase reached a maximum of about (324.8 U/mL) on the third day, and then remained stable between (223.6–313.1 U/mL) (Figure 2B). This early peak supports the role of xylanases in disrupting hemicellulose barriers to facilitate subsequent cellulose attacks and is consistent with studies on microbial consortia degrading rice straw and other lignocellulosic substrates [33]. During the entire degradation process, the enzyme activities of lignin peroxidase and manganese peroxidase remained relatively stable, suggesting sustained oxidative capacity. On the first day, they were 131.4 and 6.3 U/mL (Figure 2C), respectively, and then fluctuated between 125.1 and 181.6, and 2.55.2 U/mL (Figure 2D), respectively, reflecting their continuous involvement in overcoming lignin recalcitrance, thus enabling other hydrolases better access to cellulose and hemicellulose fractions [34].

The enzymatic activity levels demonstrated indicate effective synergistic degradation by the microbial consortium SXJG15. Such enzymatic cooperation is critical in biotechnological applications, including efficient biomass conversion for biofuel production and other value-added chemicals [30,33]. The community composition was consistent with the enzymatic activity profiles. The dominance of *Cellulomonas* and *Sphingobacterium* coincided with the highest cellulase and xylanase activities, supporting their established roles in cellulose and hemicellulose degradation. The presence of *Azospirillum* may further enhance overall activity by contributing to nitrogen fixation and promoting microbial interactions. These findings indicate that the observed enzyme production reflects the functional potential of the dominant taxa, linking microbial diversity directly to the degradation efficiency of SXJG15.

### 3.4. Changes in Microbial Community Structure

#### 3.4.1. Diversity of Microbial Communities

**Figure 3:**
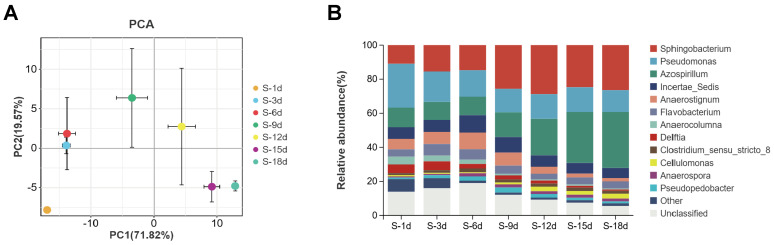
PCA plot (**A**) and microbial population structure (**B**) of the consortium SXJG15 at different stages of straw degradation. S-1d, S-3d, S-6d, S-9d, S-12d, S-15d, and S-18d represent straw degradation samples after 1, 3, 6, 9, 12, 15, and 18 days of cultivation, respectively.

Principal component analysis (PCA) was used to analyze the differences between the groups. As shown in (Figure 3A), the distances between the S-3d group and the S-6d group, and between the S-15d group and the S-18d group were relatively close, indicating that there were only slight differences in the microbial community structures between the 3d and 6d of culture, and between the 15d and 18d of culture. The distances between the S-1d group and the other groups were relatively distant, indicating that the species composition and relative abundance of the samples varied significantly with cultivation.

These temporal clustering patterns are consistent with long-term freshwater microbiome studies showing that samples collected at nearer time points cluster more tightly in ordination space, whereas those separated by longer intervals diverge [35]. These temporal clustering patterns are consistent with longitudinal microbiome studies, where samples collected at shorter intervals group more tightly in ordination space, whereas those separated by longer intervals diverge [36].

#### 3.4.2. Bacterial Composition and Relative Abundance

Microbial diversity analysis found that at the phylum level, the microbial composition of the SXJG15 community was relatively simple, mainly including *Proteobacteria*, *Bacteroidota*, *Firmicutes*, and *Actinobacteriota*, and *Proteobacteria*, *Bacteroidota*, and *Firmicutes* were the dominant bacteria. As the culture time of the consortium SXJG15 increased, the relative abundance of *Proteobacteria*, *Bacteroidota*, and *Actinobacteriota* increased significantly, while the relative abundance of *Firmicutes* decreased. At the genus level, the main SXJG15 bacterial communities included *Sphingobacterium*, *Azospirillum*, *Pseudomonas*, *Anaerostignum*, *Flavobacterium*, *Delftia*, *Anaerocolumna*, *Pseudopedobacter*, *Clostridium_sensu_ stricto_8*, *Cellulomonas*, *Anaerospora*, etc. Their relative abundance ranged from 78.7% to 92.9% (Figure 3B). Among these, the relative abundance of *Sphingobacterium* accounted for a large proportion, ranging from 48.2% to 72.1%.

By comparing the genus level within the groups at different culture time periods, the relative abundance of *Sphingobacterium* increased with the extension of culture time, from 10.9% at 1 day to 26.4% at 18 days, but the relative abundance was the highest at 12 days, about 28.8%. The relative abundance of *Azospirillum* remained between 10.6 and 11.5% from 1d to 6d, and then increased rapidly to reach 32.9% after 18d (Figure 3B). The relative abundance of *Pseudomonas* decreased with the extension of culture time. Studies have shown that *Sphingobacterium* can not only secrete cellulose and other enzymes to directly degrade cellulose, but also use intermediates such as acetic acid and lactic acid to adjust the pH of the fermentation system to maintain a neutral level to coordinate the degradation of straw [37,38,39]. Moreover, *Azospirillum* N-fixation [40] likely counteracted acidification (Figure 1A), enabling sustained enzyme activity. *Azospirillum* can fix atmospheric nitrogen [40], and it can be combined with other lignocellulose degrading bacteria to improve cellulose degradation and nitrogen fixation efficiency [41,42]. The detection of *Azospirillum* within SXJG15 indicates a potential for biological nitrogen fixation that may partly balance nitrogen limitation and support sustained enzyme production during decomposition. Although nitrogen balance was not quantified, this trait suggests an intrinsic advantage of the consortium for stable straw degradation under field conditions.

### 3.5. Diversity of Enzymes Responsible for Lignocellulose Degradation in Microbial Communities

**Figure 4:**
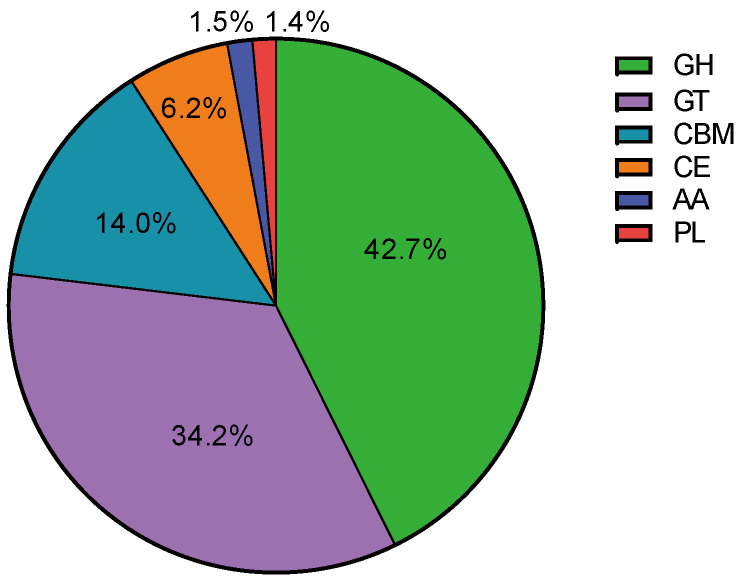
Proportion of carbohydrate-active enzyme families in the microbial flora.

**Figure 5:**
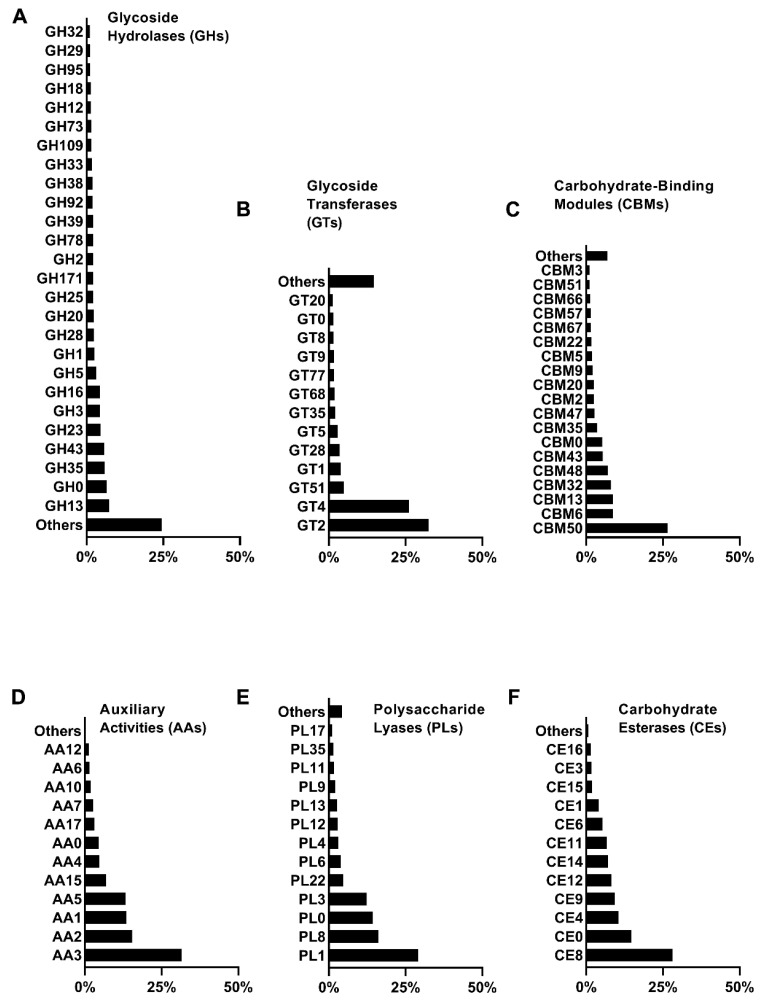
Relative abundance of CAZy enzyme families involved in lignocellulose degradation. (**A**) Glycoside Hydrolases; (**B**) Glycoside Transferases; (**C**) Carbohydrate-Binding Modules; (**D**) Auxiliary Activities; (**E**) Polysaccharide Lyases; (**F**) Carbohydrate Esterases.

In order to investigate the metabolic capacity of microbial communities for lignocellulose degradation, the diversity of carbohydrate-active enzymes present in the microbial communities was analyzed using metagenomics data from the CAZy enzyme database. The lignocellulose-degrading enzymes annotated in the metagenomics sequences belong to six CAZy enzyme groups: glycoside hydrolases (GHs), glycosyltransferases (GTs), carbohydrate-binding modules (CBMs), carbohydrate esterases (CEs), polysaccharide lyases (PLs), and auxiliary activity enzymes (AAs). GHs accounted for the highest proportion (42.7%), followed by GTs (34.2%), CBMs (14.0%), CEs (6.2%), AAs (1.5%), and PLs (1.4%) (Figure 4), indicating that the SXJG15 microbial consortium had great potential in utilizing lignocellulose and its derivatives. GH, GT, and CBM (Figure 5A–F) are the main functional enzymes involved in carbohydrate metabolism [43].

The degradation of cellulose is accomplished by the synergistic action of endo- and exoglucanases. Endoglucanases (EC 3.2.1.4) randomly attack the amorphous regions of the cellulose chain, generating new ends for further hydrolysis, while exoglucanases act on the reducing ends (EC 3.2.1.176) or non-reducing ends (EC 3.2.1.91) to generate long-chain oligosaccharides. A total of 134 GHs (including the unclassified GH0 family) were identified, of which 25 (excluding GH0) accounted for more than 1%, among which GH1, GH3, and GH5 families were mainly responsible for cellulose degradation, and GH43, GH3, GH16, GH5, GH1, GH2, GH39, and GH12 were related to the degradation of hemicellulose (Figure 5A).

In addition, GH13, GH35, GH78, GH92, GH38, GH95, and GH29 families were mainly composed of α-glucosidases, β-galactosidases, β-glucosidases, α-mannosidases, and α-fucosidases, which played a vital role in the hydrolysis of lignocellulose. Overall, our study found that GH families involved in hemicellulose degradation were more abundant than those involved in cellulose degradation, which is consistent with the higher degradation rate of hemicellulose. In the CAZyme family, 17 CE and 13 AA families were identified, respectively (including unclassified CE0 and AA0). CE4, CE12, CE6, CE1, CE3, and CE16 (Figure 5F) act on the ester bond between the sugar group and the side chain groups such as acetic acid or ferulic acid, promoting the deacetylation of hemicellulose, thereby enhancing the effect of GHs. AAs cover a series of oxidoreductases responsible for the degradation of insoluble polymers such as lignin and polysaccharides.

The study found that the relative abundance of the AA family was low, only 1.5%, but despite this, 40.7% lignin degradation was achieved, suggesting GH-AA synergy (e.g., GH5 disruption of lignin-carbohydrate complexes enhancing AA2 peroxidases access) [44]. This mirrors genomic heterogeneity in non-homologous lignocelluloses (PDF1). The family related to lignin degradation was identified, including AA3, AA2, AA1, AA5, AA4, AA10, and AA6. AA2 and AA1 (Figure 5D) represent manganese peroxidase, lignin peroxidase, multifunctional peroxidase, and multi-copper oxidase, all of which are involved in lignin degradation. Their relative abundances in the AA family were 15.4% and 13.4%, respectively. AA3, AA5, AA4, AA7, and AA6 represent GMC oxidoreductase, vanillyl alcohol oxidase, benzoquinone reductase, and oligosaccharide oxidase, respectively.

Although they are not directly involved in the decomposition of lignin, they can produce essential metabolites required by heme peroxidase. PLs can cleave glycosidic bonds. Although the relative abundance of PLs was only 1.4%, the detection of MnP and LiP activities suggests auxiliary oxidative mechanisms within the consortium. Although classical ligninolytic fungi were not detected, the MnP and LiP activities may arise from bacterial members of the consortium, particularly *Cellulomonas* and *Sphingobacterium*, which have been reported to produce peroxidase-like enzymes capable of lignin oxidation. These bacterial peroxidases likely contribute to the observed lignin depolymerization under aerobic conditions. The above results showed that the bacterial community screened in this study contained a highly enriched rice-straw-degrading enzyme system, composed of a variety of hydrolases and non-hydrolases that could effectively degrade the rice straw matrix.

### 3.6. Contribution of Dominant Bacteria to GH Involved in Lignocellulose Degradation

To better understand the potential functional contributions of the major microorganisms identified in the microbial community degrading rice straw, the dominant bacterial groups and their GH abundances were analyzed based on the annotation data of metagenomics sequencing. The abundance of GH-encoding genes varied among the top 10 dominant bacterial phyla. It is noteworthy that *Sphingobacterium* and *Cellulomonas* were the main bacterial groups in terms of overall abundance and diversity of GH, followed by *Clostridium*, *Brevumdimonas*, *Paracticibacter*, *Pseudomonas*, *Azospirillum*, *Flavobacterium*, *Paracticibacter*, and *Parapedobacter*. GH10 and GH11 are the enzyme families most closely involved in hemicellulose degradation [45]. *Cellulomonas* accounted for 67.72% of GH11 ***, and *Sphingobacterium* and *Cellulomonas* accounted for 66.02% and 18.42% of GH10, respectively. GH5 and GH9 enzyme families were enriched for cellulose degradation enzymes [46,47]. *Sphingobacterium* and *Cellulomonas* accounted for 56.86% and 10.52% of GH5, respectively. *Sphingobacterium* was the most important contributor to the abundance of GH9 (86.66%).

Moreover, *Sphingobacterium* contributed 86.66% to GH9 (exoglucanase), correlating with its large-scale dominance (Figure 3B) and confirming its role in crystalline cellulose breakdown. In addition, in the genus *Sphingobacterium*, the abundance of genes encoding enzymes such as GH13, GH92, and GH38, which are composed of oligosaccharide degrading enzymes [47], exceeded 50%, and GH35 and GH29 were even above 90% (Table 1). The exceptional xylanase activity (324.8 U/mL) aligns with metagenomics GH10/GH11 abundance and exceeds *Trichoderma harzianum* (81.2 U/mL) [14], likely due to *Sphingobacterium*-*Cellulomonas* synergy.

These data indicate that *Sphingobacterium* and *Cellulomonas* are the main genera involved in the degradation of hemicellulose and cellulose. Specifically, *Cellulomonas* dominated hemicellulose-targeting GH11 (67.72%), while *Sphingobacterium* drove cellulose-hydrolyzing GH5/GH9 (56.86–86.66%), revealing a division of labor absent in LTF-27 (PDF2). In addition, *Clostridium*, *Brevumdimonas*, *Paracticibacter*, *Pseudomonas*, *Azospirillum*, *Flavobacterium*, *Paracticibacter*, and *Parapedobacter* also have some homologs of the carbohydrate active modules of GHs, with abundances ranging from 0.11% to 17.13% (Table 1), indicating that these bacteria are also involved in the hydrolysis of lignocellulose, oligosaccharides, polysaccharides, and cellobiose. The CAZyme diversity in SXJG15, including GHs, PLs, and peroxidase-like AAs, underlies the observed hydrolysis rates, reflecting a synergistic enzymatic system that efficiently degrades cellulose, hemicellulose, and partially lignin under mesophilic conditions.

Our data also provide evidence for a potential link between microbial taxa and their functional traits. Here, the characterized microbial consortium efficiently degraded rice straw. This process was attributed to the synergistic effect of two dominant bacteria, *Sphingobacterium* and *Cellulomonas*, as well as contributions from multiple other bacteria. The functional (CAZyme) composition of these bacteria was strongly correlated with the structure of the dominant species in the microbiota.

## 4. Conclusions

**Figure 6:**
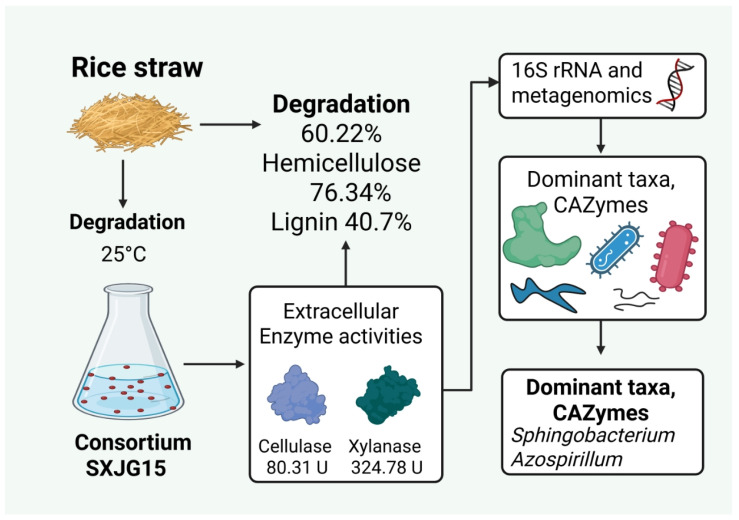
The SXJG15 consortium degraded rice straw with a (60.22%) biomass loss, exhibiting high cellulase and xylanase activities, and metagenomics analysis identified *Sphingobacterium* and *Azospirillum* as CAZyme-rich taxa.

In nature, the complete degradation of straw is the result of the interaction of many microorganisms. In this study, the microbial consortium SXJG15 was shown to secrete multiple enzymes to synergistically degrade the lignocellulose in straw. The composition diversity and richness of SXJG15 at different stages of straw degradation were significantly different, with *Sphingobacterium*, *Azospirillum*, *Pseudomonas*, and *Cellulomonas* being the dominant functional genera. SXJG15 achieves 52.5% straw degradation at 25 °C within 18 days. The synergy between *Sphingobacterium* (cellulose/GH9) and *Cellulomonas* (hemicellulose/GH11) provides a template for mesophilic moderate temperature (25 °C) consortium design. Field trials should validate its ability to reduce decomposition time by 50% versus natural decay while mitigating root rot risk. In summary, this study provides a theoretical foundation for understanding the degradation mechanisms of SXJG15 and supports its application in the bioconversion of straw biomass for sustainable agriculture (Figure 6).

## Figures and Tables

**Table 1:** Analysis of the contributions of dominant species in microbial consortia to GHs for lignocellulosic degradation.

	*Sphingobacterium*	*Clostridium*	*Cellulomonas*	*Brevumdimonas*	*Pararcticibacter*	*Pseudomonas*	*Azospirillum*	*Flavobacterium*	*Parapedobacter*	*Pseudopedobacter*
GH13	51.26%	11.50%	12.45%	7.15%	1.83%	4.14%	3.05%	0.84%	0.96%	1.00%
GH35	90.63%	0.66%	1.07%	0.79%	2.89%	0.26%	0.11%	0.00%	1.53%	0.96%
GH43	75.93%	3.47%	5.92%	0.81%	3.20%	2.35%	2.01%	0.20%	1.38%	1.43%
GH23	58.49%	8.43%	7.82%	7.65%	0.90%	3.73%	3.56%	1.85%	1.04%	0.98%
GH3	66.04%	7.88%	8.48%	3.17%	2.10%	2.39%	2.10%	1.86%	0.30%	1.27%
GH16	83.83%	0.15%	4.16%	0.61%	3.15%	0.60%	0.49%	0.69%	1.65%	0.98%
GH5	56.86%	5.86%	10.52%	8.02%	2.14%	6.10%	3.58%	0.56%	0.38%	0.63%
GH1	45.26%	17.13%	17.65%	3.47%	2.16%	2.58%	1.79%	1.65%	0.75%	1.20%
GH28	55.65%	12.70%	7.59%	7.98%	3.41%	3.14%	1.45%	1.05%	1.18%	1.97%
GH2	70.45%	2.89%	5.71%	6.40%	5.56%	1.43%	0.72%	0.00%	1.10%	1.49%
GH39	73.08%	6.27%	3.16%	2.56%	1.80%	0.69%	1.02%	0.00%	1.69%	1.22%
GH92	89.39%	1.83%	1.54%	1.63%	2.21%	0.21%	0.32%	0.00%	1.20%	0.44%
GH38	54.85%	13.81%	5.57%	7.33%	2.27%	1.97%	1.65%	1.61%	1.94%	1.12%
GH12	57.88%	12.36%	10.81%	5.22%	1.42%	1.00%	2.73%	0.00%	0.54%	0.37%
GH29	94.39%	0.23%	0.00%	0.00%	2.73%	0.00%	0.00%	0.00%	1.15%	1.15%
GH32	76.87%	6.27%	7.16%	0.00%	2.68%	1.34%	0.00%	0.00%	0.74%	0.27%
GH10	66.02%	2.88%	18.42%	5.76%	1.04%	1.06%	0.41%	0.00%	0.75%	1.34%
GH11	0.00%	2.06%	67.72%	0.00%	9.76%	0.00%	0.00%	0.00%	0.00%	10.72%
GH9	86.66%	1.37%	6.19%	0.00%	0.41%	0.46%	0.00%	0.00%	1.69%	0.00%

## Data Availability

The *16S RNA* and metagenome sequencing data were deposited in NCBI under accession numbers PRJNA1298514 and PRJNA1298566, respectively.
